# First-time couples’ shared experiences of the birth environment

**DOI:** 10.18332/ejm/153946

**Published:** 2022-10-27

**Authors:** Rebecca Mizzi, Rita Pace Parascandalo

**Affiliations:** 1 Mater Dei Hospital, Ministry of Health, Msida, Malta; 2 Department of Midwifery, Faculty of Health Sciences, University of Malta, Msida, Malta

**Keywords:** birth environment, birth experience, labor and birth, mothers experiences, father experiences, joint or shared experience

## Abstract

**INTRODUCTION:**

This study aimed to explore both mothers’ and fathers’ lived experiences of the birth environment. Objectives were set to explore how the physical, psychosocial, spiritual and cultural environment during labor, influence the parents’ birth experience, and to delve into the similarities and differences between mothers’ and fathers’ views and experiences of the birth environment.

**METHODS:**

The study adopted an interpretive phenomenological research design. A purposive homogenous sample of seven couples was recruited from the main local public hospital in Malta. Data were collected using one-time, face-to-face, semi-structured interviews with each couple. The birth territory theory by Fahy guided this study and interpretive phenomenological analysis was used to analyze, interpret and elicit the meanings that participants attributed to their experiences of the birth environment.

**RESULTS:**

Three super-ordinate themes emerged from the data: ‘The home–hospital gap’, ‘Midwifery care’ and ‘Movement in labor’. A conflict between the comfort of home and home-like aesthetics, and the reassuring, but foreign, clinical environment and medical equipment, was felt by mothers and fathers. The midwife was a fundamental part of the birth environment for the parents, taking precedence over the physical environment. Movement in labor was important to mothers while fathers became more involved when mothers were mobile during labor. The birth environment consisted of facilitating and impeding factors to movement, which made an impact on the parents’ experiences.

**CONCLUSIONS:**

Mothers and fathers experienced the birth environment from different perspectives. However, they have indicated similar needs and desires from the birth environment, creating a shared experience.

## INTRODUCTION

The birth experiences of both mothers and fathers, have long lasting effects on themselves and their families^1^. Where a mother gives birth is crucial to her experience, health, wellbeing and progress in labor^2^. We often forget that fathers also have their own needs from the birth environment, which will influence their ability to support the mother and impact their own experience of childbirth^3^. Following a literature review, it became clear that, evidence on mothers’ and fathers’ joint experience of the birth environment, as couples, is scarce. To address this gap in the literature, this study aimed to explore both mothers’ and fathers’ lived experiences of the birth environment. The literature review also presents a clear consensus that birth environment goes beyond the physical space, and is influenced by those present, the institution and holistic setting^4-6^. Hence, this study goes beyond the physical perspective of the birth environment to include all aspects.

The study objectives were to explore how the physical, psychosocial, spiritual and cultural environment during labor, influence the parents’ birth experience and to delve into the similarities and differences between mothers’ and fathers’ views and experiences of the birth environment. Couples’ experiences of the birth environment during early labor when at home, and experiencing labor at an obstetric-led public hospital for those having a normal vaginal birth, were studied.

## METHODS

### Study design

The research study sought to elicit meaning to couples’ experiences of the birth environment. The study adopted an interpretive phenomenological research approach, using interpretative phenomenological analysis (IPA) to analyze the data^7^. The birth territory theory guided this research study throughout, from its design through to the interpretation of the results. The birth territory theory is made up of two main components: the physical terrain, and jurisdiction. The physical terrain is described using two opposites: the ‘surveillance room’, and the ‘sanctum’. The ‘surveillance room’ lacks privacy and optimizes professionals’ supervision. The ‘sanctum’ birth space provides a home-like and private environment, optimizing physiology^8^. Jurisdiction refers to the use of power, by mothers or others within the birth space. Redesigning the birth territory to enhance a mother’s integrative power improves satisfaction and empowers mothers. This power can be facilitated by the midwife through midwifery guardianship. In contrast, disintegrative power separates the mother from her inner power to birth intuitively. This theory takes a holistic approach towards the birth environment and embraces the individuality of each mother and her labor, as well as the importance of the midwife as part of the birth environment^8,9^.

### Setting

The birth environment is a culturally and geographically sensitive area of research^6^. Participants for this study were recruited from the main tertiary general public hospital in Malta. This hospital has one obstetric-led maternity unit, with midwives attending all normal vaginal births, and obstetricians called when complications arise. The unit consists of individual birthing rooms with ensuite facilities and a bed at the center of the room. Home birth and midwife-led units are not available as part of the national health services in this setting. This was the first study which explored couples’ experiences of such a birth environment in this hospital.

### Participants

A purposive homogenous sample allowed for focus within the study, data saturation was used to guide the number of couples in the sample, which led to a sample of seven couples. Parents meeting the eligibility criteria were approached with an information letter for recruitment by an intermediary person, a midwife, who was not involved in this research project. Data were collected in early 2017 and written informed consent was obtained prior to each interview from participants.

First-time parents were included in this study, to exclude the influence of previous birth experiences. The researchers were interested to know about the complete experience of the birth environment, to include both the home environment in early labor and the hospital environment where labor progressed and birth occurred for the participants. Therefore, only couples who came to the hospital in spontaneous labor were included in the study. To better understand the parents’ experience of the home environment during early labor, only couples who lived in the same household were included in the study, as the home environment was a shared environment for each couple. Additionally, only couples whose child was born at term by normal vaginal birth were included. Couples experiencing induction of labor, instrumental delivery or cesarean section were excluded from the research study. Couples whose infant required neonatal intensive care and mothers who experienced complications during or after birth were excluded. Couples where both mother and father were over 18 years of age were included in the study. Couples where either mother or father were unable to communicate in Maltese or English, were not included in the study.

### Data collection and analysis

One-time, face-to-face semi-structured interviews were used for data collection in this study. The interview schedule consisting of four open-ended questions, asked couples about their experiences of the physical, psychosocial, cultural and spiritual birth environment and how the environment made them feel. Participants were also asked what they would change from the birth environment they experienced. Such questioning allowed participants to reflect on the various aspects of their birth environment and not only on the physical dimension. Demographic data collected included the participants’ age, nationality, attendance to antenatal education and age of the baby at the time of the interview. The interview schedule was self-designed by the researchers, following a thorough review of relevant literature.

Interviews were carried out by one researcher (RM), following the mothers’ and their babies’ discharge from the hospital, within the first four weeks postpartum. Each couple participated in a joint interview, to capture their joint experience of the birth environment.

A pilot study of two interviews was carried out and no necessary changes to the interview schedule were deemed necessary. The main study consisted of seven coupled interviews. Interview schedules were designed in both Maltese and English, allowing participants to choose their preferred language. Interviews were held in the participants’ home as this was preferred by the participants. Interviews were audio-recorded with the participants’ permission. Audio-recordings of interviews were transcribed verbatim for analysis.

Data were analyzed in the original language used during the interviews to avoid losing meaning in translation. Transcripts were read and re-read by both researchers (RM, RPP), allowing dwelling in the data and eliciting the meanings participants attributed to their experiences. Descriptive, linguistic and conceptual comments were analyzed for their meanings to the participants. Emergent themes were then developed from the exploratory notes, moving from a descriptive process to interpretation of the data. Connections across emergent themes were identified to produce a structure, recognizing the most important and essential aspects of the data. The above steps were repeated for each transcript. Patterns across the different cases were identified, which lead to a reconfiguration of themes and super-ordinate themes. Researcher triangulation was adopted by both researchers to validate the emergent themes^7^ (Table 1).

**Table 1:** Super-ordinate themes and main themes, 2017, Malta (N=7)

*Super-ordinate theme*	*Themes*
The home–hospital gap	The comfort of home
	The clinical environment
	Functional vs personal
	Privacy for an intimate moment
Midwifery care	Decision-making
	Midwives for both parents
	Midwives for midwives
Movement in labor	Facilitating a natural birth
	Feeling constrained
	The bed and alternatives

### Ethics

Ethical approval to carry out the research study was granted by the Faculty of Health Sciences Research Ethics Committee of the University of Malta, after obtaining the necessary institutional permissions allowing access to the participants.

## RESULTS

### Demographic data

Mothers’ ages ranged from 27 to 38 years. Fathers’ ages ranged from 27 to 41 years. All participants were of Maltese nationality and had given birth between one and four weeks prior to the interview. All participants had attended antenatal education sessions together as a couple. This is relevant to the study as the antenatal education program provided by the hospital includes a visit to the birthing unit and birth rooms which were later used by the participants.

### Super-ordinate themes

Data analysis led to three super-ordinate themes. ‘The home–hospital gap’ which reflects what the home and hospital environment meant to the participants. ‘Midwifery care’, where midwives were considered to be fundamental to the birth environment and ‘Movement in labor’ which takes a holistic approach to facilitating and impeding factors for movement and what these meant for the couples (Table 1).

### The home–hospital gap

The participants’ narratives started with the ‘comfort of home’ theme (Table 2). Home was seen as a comfortable and optimal place for early labor, which had a stark contrast with the clinical environment of the hospital. The couples described the clinical environment as being ‘cold’, reflected in the second theme ‘the clinical environment’. Mothers and fathers felt that the hospital environment was a foreign environment however, this environment was considered to be the social and cultural norm for childbirth in the local context:

**Table 2:** Super-ordinate theme 1: The home–hospital gap, 2017, Malta (N=7)

*Example from a verbatim quote*	*Emergent themes*	*Main theme*	*Super-ordinate theme*
***‘… at home basically I just kept doing things like normal, then we went walking.’*** (Sarah, Couple 3)	Continuing the normal day to day routine Keeping birth natural Staying comfortable	The comfort of home	The home-hospital gap
***‘One thing that always strikes me when I get to any room in hospital is that they are all very cold, it's probably the furniture because there's no woods it's all grey and blue, the environment is cold per se, which I guess is the normal environment of the hospital.’*** (Ben, Couple 1)	A cold environment Expected environment Hospitals related with ill health Presence of equipment Cleanliness and sterility Not home-like	A clinical environment	
***‘They try as much as possible to help you personalize it, I mean but at the end of the day, it's still I mean, hospital chairs, equipment everywhere.’*** (David, Couple 4)	Unfamiliar environment Equipment as daunting but reassuring Does the job Attempting to personalize the space	Functional vs personal	
***‘As soon as the doors open people look at you on the bed, maybe you are half dressed maybe you are trying to give birth. From a design point of view it makes sense that when the door opens there is an anteroom or something that you can't directly see the patient but you have to go around it, a wall, a piece of furniture, a paravento, but the fact that everyone is passing by, the door does not close properly if I remember well and as soon as someone opens the door they look at you, from my point of view maybe she won't agree with me, you feel too much exposed. At that point it's an intimate moment.’*** (Adam, Couple 2)	Intimate moment Staff attitudes around privacy Fear of breach of privacy Lack of a private physical environment	Privacy for an intimate time	

*‘At the same time you need that stuff ... there's always going to be that gap, between having a delivery at home and a delivery at hospital, you're giving up your personal space for equipment that will be there to help you in case of an emergency.’* (David, Couple 4)

A conflict between the medicalized surroundings providing reassurance and a safe environment for birth, yet at the same time being unfamiliar, was felt by the participants. There was a contrast between functionality and personalizing the birth space as seen in the theme ‘functional vs personal’. The heavy presence of equipment such as the neonatal resuscitaire, added to the clinical and functional feel of the birth room. However, the birth space was personalized by some of the participants, with the use of music and dimming the lights. Being able to personalize the environment provided familiarity and psychological reassurance particularly for mothers. Mothers and fathers felt differently about personalizing the birth space. This was more important and achievable for mothers, while fathers felt that the space was more about functionality.

A physically, but also psycho-social private space was of prime importance to both mothers and fathers. The theme ‘privacy for an intimate moment’ relates to participants’ psycho-social and spiritual need for intimacy during birth. Both the room design and professional attitudes were reflected on in this theme. The couples felt there were positive aspects, like individual birthing rooms and ensuite facilities. However, having swinging-style doors compromised privacy. Experiences of professionals’ attitudes varied greatly around maintaining privacy. Entering the room with prudence was greatly appreciated by couples:

*‘A midwife came in to talk to the midwife taking care of me, not immediately coming in, she sort of stayed by the door and when she saw that everything was OK she came in.’* (Marie, Couple 5)

### Midwifery care

Midwifery care was considered to be more important to the couple than the physical environment, emphasizing the holistic nature of the birth environment (Table 3). The midwife was seen as an essential source of information in the theme ‘decision-making’, with most of the couples having a positive experience when being provided with all their options during labor. However, not all couples were satisfied with the information they received from the midwife. Informed choice and being empowered to make one’s own choices were very important to the couples.

**Table 3:** Super-ordinate theme 2: Midwifery care, 2017, Malta (N=7)

*Example from a verbatim quote*	*Emergent themes*	*Main theme*	*Super-ordinate theme*
***‘Every step of the way I knew what was happening, she explained everything, she always gave me a choice, I choose if I want this, it's my choice not her choice.’* (Sarah)** ***‘She gave us a choice.’*** (Samuel) (Couple 3)	Information Choice Source of knowledge Patronizing attitudes	Decision-making	Midwifery care
***‘I was just standing there close to her waiting for the midwife to tell me what to do and every time she said go in front of her and she will hold on to you and I did what she said, then when she was getting tired I was telling her just last few pushes.’*** (Ben) ***‘…the midwife was asking him to be more encouraging.’*** (Claire) ***‘…but she got me really involved like.’*** (Ben) (Couple 1)	Involving the father Characteristics Forming a relationship Underestimated role Source of comfort	Midwives for both parents	
***‘…they stay calm between themselves and don't make you feel any pressure. There's a nice environment, they work really well together.’*** (Matthew, Couple 5)	Team work Attitudes between themselves Handover Backup Safe environment	Midwives for midwives	

The couples felt that the midwife’s main concern should be the mother and baby. However, when midwives encouraged fathers to be involved during labor, both mothers and fathers benefitted, as revealed in the theme ‘midwives for both parents’:

*‘I was just standing there close to her waiting for the midwife to tell me what to do and every time she said go in front of her and she will hold on to you and I did what she said, then when she was getting tired I was telling her just last few pushes.’* (Ben)

*‘The midwife was asking him to be more encouraging.’* (Claire)

*‘…but she got me really involved like.’* (Ben) (Couple 1)

Mothers and fathers differed in that fathers observed closely how the midwives worked, which put them at ease. On the other hand, for mothers it was more about how the midwife made them feel, in what was considered to be a sacred time for the mother as she focused on her labor and trusted her midwife. Mothers felt positively about how the midwives made them feel during labor with their warmth making up for the cold and clinical environment. Midwives were viewed as being calm, with the participants and their colleagues. The theme ‘midwives for midwives’ reflects upon how couples felt there was team work between midwives which made couples feel comfortable and safe within the birth environment:

*‘They stay calm between themselves and don't make you feel any pressure. There is a nice environment, they work really well together.’* (Matthew, Couple 5)

### Movement in labor

Movement in labor was related to various facilitating and impeding physical and sociocultural factors within the birth environment (Table 4). The midwife’s practice, and mother’s choices and preconceptions, were two main factors that influenced the mother’s ability to move freely during labor. For the fathers, movement and upright postures had the added advantage of keeping them involved in the labor, which in turn was satisfying and appreciated by the mothers.

**Table 4:** Super-ordinate theme 3: Movement in labor, 2017, Malta (N=7)

*Example from a verbatim quote*	*Emergent themes*	*Main theme*	*Super-ordinate theme*
***‘I think they (using different positions) help to hasten the delivery because when I was standing up gravity helps so I was standing up holding onto Ben and squatting down so I was helping, gravity was helping me.’*** (Claire, Couple 1) ***‘…what I found out was the more you move the more you make it natural despite everything the better it was for me.’*** (Daniela, Couple 4)	Upright postures Mobility to cope with contractions Involving the father Avoiding augmentation Availability of space	Facilitating a natural birth	Movement in labor
***‘…cause at first they used the wired connections but then eventually they moved to wireless but in my opinion they should have just gone for wireless immediately rather than you being constrained.’*** (David, Couple 4 )	Cardiotocography monitoring Analgesia Midwifery care	Feeling constrained	
***‘…you feel the pain in bed and you can't move, I felt more comfortable on the ball.’*** (Nicola, Couple 6)	Availability of birthing aides Expectations of second stage Staying off the bed in first stage Comfort for the mother	The bed and alternatives	

Most of the couples felt that mobility and upright postures kept birth as natural as possible, in the theme ‘facilitating a natural birth’:

*‘…what I found out was the more you move the more you make it natural, despite everything, the better it was for me.’* (Daniela, Couple 4)

The participants felt that the size and spaciousness of the delivery room facilitated movement, a positive aspect of the physical environment, together with the availability and use of birthing aides, such as the birthing ball. Participants perceived that these aided spontaneous labor and reduced the sensation of pain.

Mothers and fathers recognized how comfort and mobility are interrelated in the theme ‘feeling constrained’. Cardiotocography and pharmacological analgesia, although deemed necessary, were not desirable aspects of the birth environment. One of the mothers used epidural analgesia, and she felt that epidural analgesia consequently constrained her movement, despite being pain-free she was not particularly comfortable. However, limited movement was not related to epidural analgesia alone, as another mother felt one fixed entonox port in the room limited her mobility:

*‘…had the gas been portable, that, because even to go to the bathroom I felt uncomfortable, because as I started to take it and started to rest as I was getting up, you have to take it beforehand so I didn't want to stand in any way and leave it behind, I was afraid to leave it behind, had it been portable you could move, as I didn't want to stand up and move because of it.’* (Anne, Couple 7)

The theme ‘the bed and alternatives’, explored the availability of birthing aides and the culture of a bed-centric birthing room. All participants felt that avoiding the bed and making use of birthing aides was advantageous in the first stage of labor. However, the bed still played a central role during the second stage of labor, with five out of the seven participants giving birth on the bed. The midwife’s suggestions and practices played an important role in adopting upright postures throughout labor. Staying on the bed for the second stage of labor was a sociocultural norm and doing anything alternative was seen as being unusual. The use of water-immersion was considered by two of the participating couples. However, the apparent lack of facilities and hospital policy did not give them the possibility to use water for birth.

## DISCUSSION

The super-ordinate themes: ‘the home–hospital gap’, ‘midwifery care’ and ‘movement in labor’ are discussed in relation to the holistic perspective of the birth territory theory as described by Fahy and colleagues^8-10^. Participants’ narratives of events reflected the shared experiences between couples, however, similarities and differences across mothers’ and fathers’ perspectives became evident and were further explored.

### The home–hospital gap

A contrast between the clinical and functional delivery room and the personal and comfortable setting of their own home emerged throughout the study. The clinical appearance and presence of equipment experienced by the participants relates to Fahy and colleagues’ birth territory theory’s description of a surveillance room^8-10^. The cold and clinical environment provided cleanliness, but was not comforting to the participants. The couples made reference to a sanctum-like birth environment as described by the birth territory theory when they refer to home-like and warm features, which would have been a desirable aspect of the birth environment^9,11^. However, equipment and medical assistance, despite being foreign and not aesthetically pleasing, were considered to contribute towards having a safe birth. The hospital environment was seen as being a foreign environment, yet providing reassurance. In contrast, according to the birth territory theory, the more a birth space is similar to the sanctum terrain, the safer and more confident the mother feels to give birth^9^. The findings suggest that certain aspects of the surveillance environment such as equipment, although daunting, were perceived as being necessary for a safe labor and birth. Similarly in the Sheehy et al.^12^ study of women’s expectations and experiences, and in the study of Davis Harte et al.^13^ on birth partner’s experiences, both report a foreign and clinical environment as providing reassurance. The conflicting meanings given to the hospital environment, in this study, are also reported in both of these Australian studies. Both the Hodnett et al.^14^ Cochrane systematic review and the Overgaard et al.^6^ prospective cohort study, found mothers were more satisfied in midwifery-led birth centers as opposed to obstetric-led units. Provision of a birth center, with sanctum-like aesthetics, could be one way to bridge the gap between home and hospital settings, as well as offering choice to expectant parents.

Home-like features, access to music, adjustable lighting and comfort aides, were reported in the Singh and Newburn^2^ large scale National Childbirth Trust (NCT) survey to be desirable aspects of the birth environment for mothers. The findings of the present qualitative study suggest similar improvements to the clinical hospital setting, with some of the participants feeling that the aesthetics reflected ill-health and did not fit the joyful experience of birth. Some mothers managed to create their own sanctum terrain with the help of their midwife. Mothers and midwives attempted to create more of a sanctum birth space, by personalizing their surrounding with the use of music and dimming the lights. For the fathers, the environment was perceived to be purely functional and not capable of being personalized. This could be related to the fact that personalizing the environment was directed at improving the mother’s comfort in labor and not the father’s wellbeing. The findings center around the mothers’ physical comfort and wellbeing. In contrast, the phenomenological delivery-room design study of Hyldgaard Nielsen and Overgaard^15^, based in Denmark, found the birth environment providing space, comfort and personal touches for the birth partner’s comfort, an important element in birth room design for mothers.

Maintaining the parents’ privacy in the intimate moments of labor and birth is a defining aspect of providing a sanctum birth space^9,10^. For both mother’s and father’s psychosocial and spiritual wellbeing, depended upon the provision of privacy to share this intimate time together, privacy needed to be maintained by the physical environment and the staff at the delivery suite. Privacy was as important to fathers as it was to mothers. This resonates with Davis Harte et al.^12^ findings, which recognized privacy as essential for birth partners. However, the culture of a hospital, which is not the participants’ own space, can never be completely private. The design of the hospital room meets the hospital’s needs, allowing hospital staff to enter and exit as needed. A change in the design of the room and presence of a screen for privacy, as recommended by a father in this study, is also a recommendation of the BUDSET (Birth Unit Design Spatial Evaluation Tool) designed by an expert panel of midwives and architects^16^. Mothers and fathers were content when their birth space was treated as a private space. Unfortunately, privacy had been invaded for some of the couples when staff, not involved in their care, entered the room without prior warning. During labor, couples wished to be surrounded only by those directly involved in their care.

### Midwifery care

The midwife was recognized to be of paramount importance in the holistic birth environment. Likewise, in the birth territory theory, midwifery guardianship optimizes the mother’s safety and respect within the birth terrain, empowering the mother for birth^8-10^. Building a rapport with the midwife was important to all participants in the present study. Most participants felt that the manner in which they were cared for was more important than the physical environment they experienced. When the midwife caring for the couple was welcoming and caring, the cold and clinical aesthetics of the birth environment did not remain as daunting. Hyldgaard Nielsen and Overgaard^15^ found that an alternate hospital birth room, with warm, healing features amplified the psychosocial and emotional support provided by midwives, who were considered an integral part of this type of environment. In the present study, with a ‘cold and clinical’ environment, the midwife still provided warmth and wellbeing towards both mothers and fathers. Likewise, Goldkuhl et al.^4^ ethnographic study found that the midwife enhancing a mother’s integrative power through her care, increases control, safety and satisfaction, beyond what the physical environment alone can provide.

Participants formed a relationship with their midwife based on trust, the midwife’s attitude, their confidence in her skills and the information she provided them. Conforming to the birth territory theory, midwifery guardianship, which facilitates the mother’s integrative power during labor and birth, is highlighted in these findings. Decision-making is an intrinsic part of midwifery guardianship^9^. The study findings demonstrate that couples preferred to be given unbiased information and explanations to facilitate making their own choices. Those couples who were not involved in decision-making were not as content with the midwifery care they received. These findings resonate with two large longitudinal studies: Rudman et al.^17^ found that not being involved in decision-making negatively impacted mother’s satisfaction in labor. Hildingsson et al.^18^ found that fathers’ experience of midwifery care was dependent on information giving and decision-making.

In the present study, midwifery care, although still primarily focused on the mother and baby, also incorporates the father. Mothers and fathers were content when the midwives involved the fathers during labor. However, not all fathers in the study had the same experience of midwifery care. When fathers were guided by the midwives, on how to be supportive, and were made to feel included in the labor, their experience was better. For the mothers this was also beneficial, as their partner offered a unique supportive role. The father’s physical, psychological and spiritual support facilitates the release of oxytocin and hence results in a physiological and satisfying birth experience^19^. Therefore, when the father was guided by the midwife on being supportive towards the mother, both mother and father had a positive experience. When midwives did not provide fathers with support, fathers felt helpless. The present study, in accordance with the mixed-methods study of Johansson et al.^3^, found that midwives need to include fathers for them to become involved during labor. The uniqueness of the present study is that exploring the couples’ joint perspectives, emphasizes how meaningful it was for the mother, when the midwife involved the father, and this facilitated a shared experience of birth.

Midwives were viewed as being calm and serene with the participants and with each other. This calm attitude influenced the environment, including the parents, to remain calm. Fathers took on an observer role during labor, whereas mothers were more concerned about how the environment made them feel on a psychological and spiritual level. Consumed in their labor, mothers did not observe much. Fathers on the other hand, were very observant of the physical and psychosocial environment. This pertained especially to midwifery care. Fathers watched how the midwives worked, especially the interactions and the team work between midwives. Likewise, fathers in the phenomenological study of Longworth and Kingdon^20^ also took on an observer role, which was associated with being vigilant of all that was going on for the sake of the mother and baby. Midwives supporting each other put couples at ease and created a safe environment.

### Movement in labor

A spacious birth room with birthing aides available to use, facilitates freedom to mobilize, improving mothers’ experiences, as seen in this study and previous research across various birth units in the United Kingdom carried out by Symon et al.^21^ and Singh and Newburn^2^. However, perceived spaciousness was not just related to the size and layout of the room, but also to the care given, which influenced the mother’s ability to utilize the space. Similarly, in the present study, factors influencing movement in labor included the midwife’s practice and the mothers’ choices.

Movement was associated with keeping labor as natural as possible. The knowledge acquired prior labor, the midwife’s advice and the participants’ own experiences during labor led them to firmly believe that mobilizing kept labor natural. For mothers, listening to their own bodies, by moving as they felt was right for them at the time, facilitated a natural birth. The findings of this study present a relationship between comfort and movement in labor. Being constrained for movement diminished a mother’s comfort in labor. Fathers encouraged mothers to mobilize in labor, particularly when the midwife also encouraged the mother to mobilize. In accordance with the birth territory theory, midwifery guardianship was present when the midwife facilitated the mother’s own inner power to listen to her body and mobilize. Disintegrative power may also be exerted with the midwife undermining the mother’s ability to listen to her body to move around freely as when cardiotocography (CTG) monitoring led midwives to tell mothers to stay in a particular position, which limited mobility. Interestingly, couples did not associate the CTG with the rest of the medical equipment, contrary to the birth territory theory which includes the use of CTG as part of the surveillance terrain^8^. CTG was a reassuring and prevalent part of the birth environment for the participants. However, CTG interfered with a mothers’ ability to move around freely and to be comfortable. For this reason, telemetric CTG was preferred to facilitate movement. This reflects the participants’ expectations and compliance to a surveillance terrain. Additionally, pharmacological analgesia was for some participants a constraining factor for mobility in labor. Dependence on pharmacological analgesia restricted the mothers’ movement and these participants would have preferred to use their chosen method of analgesia and be able to move freely, for a more comfortable labor and improved experience.

The benefit for fathers, in the present study, was that upright postures gave them an active role in the labor. The mixed-methods study of Johansson and Thies-Lagergren^22^ also found that fathers had a more positive experience when their partner used an upright birth position. As seen in the present study, this was related to having a more useful role during birth as opposed to other birth positions. Involving the father with the mother’s upright postures and mobility was also meaningful to the mothers.

Mobility and upright postures were very popular with the participants during the first stage of labor. However, the bed remained a prominent part of the second stage of labor. Not all mothers were comfortable with adopting different positions during the second stage, as the supine position was seen as the sociocultural norm. The BUDSET tool also acknowledges the influence of culture and healthcare practices on the use of the bed and supine position for birth. The presence of birthing aides such as the birthing ball, birthing stool and mats, in the delivery room, encouraged participants to make use of these facilities for movement in labor. The BUDSET tool recommends birthing aides being available, but does not recommend keeping such equipment available in each room^16^. On the contrary, the present study found that the presence of birthing aides in the room, as well as midwives’ suggestions to use birthing aides, encouraged their use. An important facility in the BUDSET, as well as in the birth territory theory’s sanctum birth terrain, is a deep bath or birth pool^8,16^. None of the mothers in the study made use of water for analgesia or for birth. Two of the participating couples expressed frustration that these facilities, which they thought could be advantageous for comfort and having a physiological labor, were not available to them.

### Strengths and limitations

The use of a qualitative phenomenological design offered depth to the findings and allowed the individual experiences of participants to be voiced in the study. The small homogenous sample included couples whose labor started spontaneously and did not encounter complications during labor. However, the experiences of couples undergoing medical interventions, would likely lead to varying results. Participants were Maltese nationals, therefore findings are influenced by the Maltese culture, and so may not be representative of the culturally diverse population giving birth in Malta. Participants were informed that they were being interviewed by a clinical midwife (RM). This may have influenced the information they felt comfortable to divulge during the interviews, especially when talking about the role of the midwife. The interpretive phenomenological approach recognizes the researchers’ pre-understandings of the phenomenon and in this study, the researchers were familiar with the midwifery practices in the same hospital environment being studied. Exploring the shared experience of the couple, added depth and meaning to the study. Carrying out semi-structured interviews with the couple together gave them the opportunity to remind each other, and contribute to each other’s birth stories and experiences of the environment. However, participants may have felt more comfortable to disclose certain information without the presence of their partner. Therefore, varying results and other valuable data, could have emerged had they been interviewed separately.

## CONCLUSIONS

Exploring the birth environment from the aspect of both parents, as a couple, presented a shared experience, giving an understanding of what each other’s presence in the birth environment meant to the participants as well as the meaning of the birth environment to both parents’ experiences. The findings highlight how fathers wish to be more involved, how important their presence and involvement is to their partner and how this can be facilitated or inhibited by the birth environment. The cultural and psychosocial aspects play a crucial role in the birth environment, as seen in this study’s findings. Hospital birth was experienced as a sociocultural norm. The hospital environment, equipment and personnel, offered reassurance despite being a foreign and daunting environment. Despite the clinical setting, the parents related the birth environment to keeping birth as natural as possible, this was especially evident in the ability to mobilize freely and make use of birthing aides. The midwife played an essential role within the birth environment, in relation to facilitating movement and upright postures in labor and creating the right psychosocial, emotional and spiritual environment.

Recommendations for improvements in practice for the physical environment include home-like features, birthing aides and telemetric CTG monitoring or intermittent fetal heart monitoring. To improve the psychosocial and spiritual environment, the researchers recommend improved privacy and continued involvement of the father. Further research into the shared experiences of mothers and fathers during childbirth as well as a mixed-methods study on the birth environment are recommended where the perspectives of midwives could also be explored.

## Data Availability

The data supporting this research are available from the authors on reasonable request.
